# Feasibility of large-scale eOSCES: the simultaneous evaluation of 500 medical students during a mock examination

**DOI:** 10.1080/10872981.2022.2084261

**Published:** 2022-06-13

**Authors:** Donia Bouzid, Tristan Mirault, Aiham Ghazali, Léonore Muller, Enrique Casalino, Nathan Peiffer Smadja, Baptiste Auber, Mathias Guerin, Charles Henri Sambet, Isabelle Etienne, Victoire De Lastours, Cécile Badoual, Cédric Lemogne, Philippe Ruszniewski, Université Paris Cité’ OSCE study group, Albert Faye, Alexy Tran Dinh

**Affiliations:** a Université Paris Cité and Université Sorbonne Paris Nord, Inserm IAME, F-75018 Paris, France; b Emergency Department, Bichat-Claude Bernard University Hospital AP-HP, Paris, France; c UFR de Médecine, Université Paris Cité, Paris, France; d Département d’hypertension artérielle, Hôpital Européen Georges PompidouAP-HP, Paris, France; e Infectious diseases Unit, Bichat-Claude Bernard University Hospital AP-HP, Paris, France; f Account executive- Higher Education- Zoom, San José, California; g Service de Médecine Interne, Hôpital Beaujon AP-HP, Clichy, France; h Service d’anatomopathologie, Hôpital Européen Georges Pompidou AP-HP, Paris, France; i Université Paris Cité, INSERM U1266, Institut de Psychiatrie et Neuroscience de Paris, F-75014 Paris, France; j Service de Psychiatrie de l’adulte, AP-HP, Hôpital Hôtel-Dieu, F-75004 Paris, France; k Service de gastro-entérologie et pancréatologie, Hôpital Beaujon AP-HP, Paris, France; l Service de Pédiatrie Générale, Hôpital Robert Debré AP-HP, Paris, France; m Département d’Anesthésie-Réanimation, Hôpital Bichat-Claude Bernard, AP-HP, Paris, France

**Keywords:** Objective structured clinical examination, innovative, digital training, medical students

## Abstract

The COVID-19 pandemic has led health schools to cancel many on-site training and exams. Teachers were looking for the best option to carry out online OSCEs, and Zoom was the obvious choice since many schools have used it to pursue education purposes. Methods: We conducted a feasibility study during the 2020–2021 college year divided into six pilot phases and the large-scale eOSCEs on Zoom on June 30th, 2021. We developed a specific application allowing us to mass create Zoom meetings and built an entire organization, including a technical support system (an SOS room and catching-up rooms) and teachers’ training sessions. We assessed satisfaction via an online survey. Results: On June 30th, 531/794 fifth-year medical students (67%) participated in a large-scale mock exam distributed in 135 Zoom meeting rooms with the mobilization of 298 teachers who either participated in the Zoom meetings as standardized patients (N =135, 45%) or examiners (N =135, 45%) or as supervisors in the catching-up rooms (N =16, 6%) or the SOS room (N =12, 4%). In addition, 32/270 teachers (12%) experienced difficulties connecting to their Zoom meetings and sought the help of an SOS room member. Furthermore, 40/531 students (7%) were either late to their station or had technical difficulties and declared those issues online and were welcomed in one of the catching-up rooms to perform their eOSCE stations. Additionally, 518/531 students (98%) completed the entire circuit of three stations, and 225/531 students (42%) answered the online survey. Among them, 194/225 (86%) found eOSCES helpful for training and expressed their satisfaction with this experience. Conclusion: Organizing large-scale eOSCEs on Zoom is feasible with the appropriate tools. In addition, eOCSEs should be considered complementary to on-site OSCEs and to train medical students in telemedicine.

## Background

Objective structured clinical examinations (OSCEs) have been used worldwide in healthcare education programs since Harden first introduced them in the 1970s[1]. Students usually undertake OSCEs as face-to-face interactions to assess competency in history taking, patient information, education, laboratory-result interpretation, clinical examination, or medical procedure. OSCEs are a fair evaluation method and are widely used for high stake exams as final-year medical ranking exams [2–4]. Students move through a circuit of stations to assess them in a standardized simulated environment. At each station, students complete a clinical task within a predetermined time in the presence of a simulated patient and an examiner[2]. The COVID-19 pandemic has severely impacted medical schools’ teaching programs and exams, including OSCEs, due to the social distancing measures that have been applied all over the world these past two years[5]. In this time-constrained context of clinical-skill training and assessment, the development of innovative digital strategies to pursue high-quality medical education while ensuring social distancing has been a key goal of many medical and other health schools[6]. Although several online OSCE (eOSCEs) experiences have been reported to be using a computer-based program [7] or generic teleconference systems [8,9], Zoom teleconferencing has been more widely used during the pandemic either for lectures[10], tutoring sessions, or for eOSCEs [4,11,12]. Many schools acquired a Zoom license, and both students and teachers were familiar with its use.

In the context of recurrent SARS-CoV-2 pandemic restrictions, the medical school of Université Paris Cité, the largest one in France with nearly 800 students per study year, quickly chose to develop these exams in digital form remotely on the Zoom platform.

Our objective was to evaluate the feasibility of a large-scale eOSCE training for undergraduate medical students conducted on Zoom and to assess both teachers’ and students’ satisfaction.

## Methods

### Study design

We conducted a feasibility study on the implementation of large-scale eOSCEs on Zoom to carry out a mock exam at Université Paris Cité’s medical school during the 2020–2021 college year. We established the following conceptual framework [13]:

- We confirmed the need for an alternative to on-site OSCE during the COVID-19 pandemic and beyond.
- We developed adequate technology.
- We tested eOSCEs on Zoom with small numbers of students.
- We conducted the large-scale mock exam and assessed the teachers’ and students’ satisfaction with an online survey.

We first compared Zoom to a French telehealth system (ORTIF). The fact that students were more familiar with Zoom, the better quality of the video, and the possibility of recording the videos for remote evaluation led to choosing Zoom over ORTIF. A pilot study phase divided into six sessions, each involving 30 students and 10 teachers, was then performed between August 2020 and June 2021 to test and validate the organization and technical aspects of eOSCE on Zoom. Students were satisfied by these tests, and for each session, the number of volunteers increased, though we could not include more students during the testing. These sessions allowed us to improve the application and anticipate the need for an alternative system to evaluate the students who were experiencing connection difficulties or were simply late.

We describe the final large-scale simultaneous evaluation on June 30^th^, 2021, a mock exam.

This study obtained the approval of the ethics committee of Université Paris Cité CER U-Paris N° 2021–96- BOUZID.

### Population

Medical students completing their fifth year at the medical school of Université Paris Cité (Paris, France) – corresponding to a prior residency of one year – were invited via email as all other exams to participate on a voluntary basis in an eOSCE training (mock exam) conducted in June 2021. This email was sent once a week to each student, three weeks before the mock exam. Medical faculty members with professional experience administered the eOSCE and were involved as examiners or standardized patients.

### Description of eOSCE stations

We proposed a circuit of three different eOSCE stations to the students. Expert teachers from the Université Paris Cité OSCE group prepared these stations carefully. Each station was evaluated by two other teachers and tested with volunteer residents. The first station dealt with gynecology and focused on history-taking skills. The second one was about addictology and evaluated both communication and history-taking skills. The third was about pediatrics and provided an image of a patient with chickenpox and considered skills involving therapeutic management strategy. None of these stations addressed any technical procedure or clinical examination skills to accommodate the digital environment and allowed for more straightforward evaluation.

### Conduct of the eOSCES on the Zoom™ platform

As a prerequisite, students and teachers had to ensure a good-quality internet connection and an operational webcam and microphone. The exam was composed of three eOSCE stations, and the 794 students were scheduled to take the exam from 8:30 a.m. to 12:30 p.m. Each pair of teachers consisting of a standardized patient and an examiner was assigned to a dedicated eOSCE station linked to a single Zoom conference room. Each eOSCE station lasted seven minutes, with one minute allocated to the student to read the scenario’s instructions beforehand. One day before the exam, students received a personalized online meeting schedule, which allowed them to click and connect to a Zoom conference room for each OSCE station at a specific time. For each station, one examiner welcomed the student while the standardized patient had their camera and microphone turned off. The examiner shared their screen with the student, who could read the eOSCE instructions for one minute. Then, the examiner deactivated their camera and microphone while the standardized patient appeared on the screen to interact with the student for the next seven minutes. The examiners used the SIDES-Theia™ online system to complete each student’s evaluation grid during the eOSCE station. At the end of the eOSCE station, the student was asked to click on the following link from their personalized online meeting schedule to access the next station. The order of the stations was different from one student to the other. Student number one performed station1, then station 2 and ended with station 3, whereas student number two started with station 2, then station 3 and ended with station 1.

During the eOSCEs, 135 concurrent Zoom rooms were active.

### The eOSCE application

Organizing large-scale eOSCEs on Zoom requires programming several Zoom meetings and sending them to both students and teachers before the exam.

We initiated a collaboration with Zoom in San Jose, California to develop an ‘eOSCE application’ to mass create Zoom meetings from a comma-separated values (CSV) file. It also retrieved the URLs, IDs, and passwords for these meetings so that they could be shared with eOSCE participants.

The eOSCE application was broken down into three parts:

1. a server part in Node.js using the Zoom™ JSON Web Token application programming interface (JWT API),
2. a ‘/ admin’ web interface using React for administration, and
3. a ‘user’ web interface using React and the Zoom™ web software development kit (SDK) to participate in Zoom videoconferences.

After the application generated all the Zoom meetings, we sent all students’ and teachers’ invitations using an Excel file integrating macros.

The eOSCE application required approximately 50 hours of development by our IT engineers, Zoom engineer, and a hired freelance developer. The medical school of Université Paris Cité’s medical school donated 10,000 euros for the project.

### SOS room ± catching-up rooms

During the eOSCEs, teachers and students were outside the medical school. For this reason, we created an assistance platform to solve any technical issues.

### SOS room

On the day of the exam, several members of the OSCE group (*n* = 14) and IT engineers (*n* = 2) were physically gathered in one of Université Paris Cité Medical School’s conference rooms, called the ‘SOS room.’ Each member oversaw 20 teachers who may potentially ask for help. The means of communication used between the member of the SOS room and the teacher in trouble was the telephone via a specific number assigned. Members of the ‘SOS room’ had two primary missions. First, they provided support to teachers connecting to their Zoom meeting in case of technical difficulty and checked that all Zoom rooms were ready to welcome students at 8:30 a.m. Second, they assisted students who reported having problems, such as difficulty connecting to an eOSCE station by using a specific homemade online document and routed students who were behind schedule to catching-up rooms.

### Catching-up rooms

Eight catching-up rooms were dedicated to students who were outside of or behind their planned schedule of connections to the different eOSCE stations. The catching-up rooms were also composed of a standardized patient and an examiner who could do the three eOSCE stations with a student who had left the initial course, regardless of the reason. The student reported being late on the specific homemade online document from Université Paris Cité. This online sheet allowed the member of the SOS room to call the student back to specify the difficulty and resolve the problem by assigning a specific connection link to a ‘catching-up room’ to let this student complete the eOSCE stations that were missing. Teachers in catching-up rooms communicated with the members of the SOS room using WhatsApp™.

### Teachers’ training sessions

Four one-hour Zoom online training sessions were organized for teachers in the week preceding the eOCEs to do the following:

- check the connection to their Université Paris Cité institutional Zoom account;
- explain the online invitation process to a dedicated OSCE station per teacher pair;
- provide a quick reminder of all of Zoom’s features regarding waiting rooms, live chat, renaming, and screen sharing, which was mandatory to show students instructions and the record button;
- explain the OSCE station scenarios and their evaluation grids; and
- remind the teachers of the use of the SIDES-Theia online student evaluation platform.

### Students’ and teachers’ feedback

We sent both students and teachers an online survey to assess their satisfaction. The students’ survey included questions on the organization process, their stress level during the three stations, and how they perceived this experience. The teachers’ survey included questions on the organization process, the training sessions, and how they also perceived this experience.

These surveys were validated by Université Paris Cité’s pedagogical commission and were tested by a panel of teachers ahead of their submission.

We also held an online debriefing on the same day via Zoom, where students and then teachers could ask any questions related to the stations or the organization of these eOSCEs.

### Outcomes

Feasibility was assessed based on these criteria: The proper functioning of the application (creation of 135 functional Zoom meetings and the successful logging and connection for all teachers) and the students’ and teachers’ satisfaction, which was assessed by both the online survey and the immediate Zoom debriefing.

## Results

### Participants

On June 30^th^, 2021, 531 out of 794 fifth-year medical students (67%) participated in this large-scale mock exam distributed in 135 Zoom meeting rooms. Two hundred ninety-eight teachers participated either in the Zoom meetings as standardized patients (*N* = 135, 45%), examiners (*N* = 135, 45%), in the catching-up rooms (*N* = 16, 6%), or the SOS room (*N* = 12, 4%). All links to the Zoom meetings generated by the eOSCE application were functional and correctly provided to the participants.

Of the 270 teachers, 32 (12%) experienced difficulties connecting to their Zoom meetings and sought the help of an SOS room member. Of the 531 students, 40 (7%) were either late to their station or had technical difficulties, declared those issues online, and could be welcomed in one of the catching-up rooms to perform their eOSCES stations. In addition, of the 531 students, 518 (98%) completed the entire circuit of three OSCE stations. Thirteen students did not join the catching-up rooms to complete the missing stations. Eleven of these students did not answer the phone. Members of the SOS rooms tried to reach them, and the two others had issues with their internet connections.

In Table 1, we provide a summary of all the requirements for successful large-scale eOSCEs.

**Table 1.** eOSCE requirements

Requirements	Uses
**OSCE application**	Makes it possible to mass create Zoom meetings from a CSV file containing teachers’ and students’ names and email addresses
**Excel + Macro (functions)**	Makes it possible to send OSCE invitations directly to teachers and students
**Incident sheet**	Allows students to declare online if they have any trouble accessing their OSCE station during the exam
**SOS room**: *(Conference room with several computers, phones, and a stable internet connection)*	The ‘SOS room,’ which includes all logistical and technical assistance during the exam helps teachers connect to their Zoom meetings and helps students catch up on their missed stations (declared on incident sheets)
**Several catching-up Zoom rooms**	Allows students to catch up on their missed stations
**WhatsApp**	Helps the SOS room and catching-up rooms communicate

### Students’ feedback

Of the 531 students, 267 (50%) connected to the Zoom debriefing meeting at the end of the exam scheduled by the organizing team.

In addition, 225 of the 531 students (42%) answered the online survey. Furthermore, 221 of the 225 students (96%) who answered the online survey highlighted that their connection to the Zoom meetings for the OSCE stations circuit was ‘easy’ or ‘very easy.’

**Figure 1.**
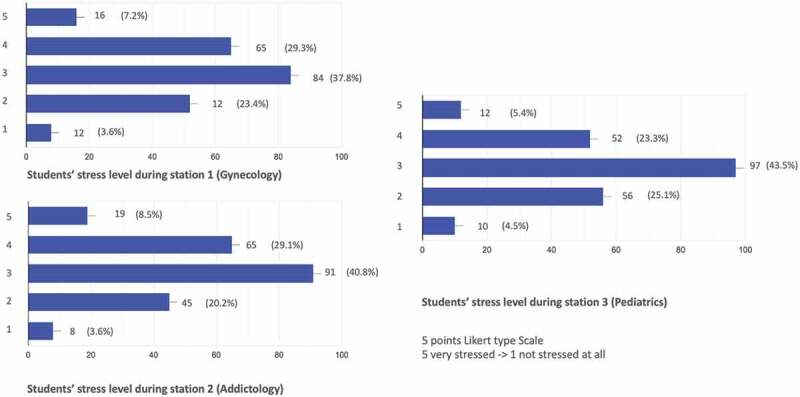
Students’ auto-evaluation of their stress level (5 points Likert type scale).

Regarding their evaluation for each of the three OSCE stations, students most often expressed a stress level of 3 on a Likert scale with 5 response alternatives: from no stress (1) to extreme stress (5) (Figure 1).

Of the 225 students, 194 (86%) found eOSCEs helpful for training and were satisfied by this experience (Table 2).

**Table 2.** Students’ and teachers’ feedback

	Students (N = 225/794)	Teachers (N = 168/270)
Absent	263 (33%)	0 (0%)
Present	531 (67%)	270 (100%)
Answered the online survey	225 (42%)	168 (62%)
Connection difficulties	40 (7%)	32 (12%)
Satisfied by the communication ahead of the eOSCEs	138 (61%)	163 (97%)
Found eOSCEs to be a suitable training method	152 (67%)	142 (84%)
Satisfied by the experience	194 (86%)	145 (86%)

Finally, 29 (13%) moderately satisfied students and 2 (1%) unsatisfied students stated that they were not prepared for online training and that they would have preferred on-site OSCEs.

In the free comments section, the students expressed their gratitude for this opportunity to train for OSCEs. While they expressed their wish to have on-site OSCEs as a sanctioning exam, they also wished for more eOSCEs for training purposes.

### Teachers’ feedback

Of the 270 teachers, 168 (62%) answered the online survey. In addition, 58 (34%), 87 (52%), and 23 (14%) were respectively ‘very satisfied,’ ‘satisfied,’ and ‘moderately satisfied’ by the experience (Table 2). The most frequent free comment reported in the survey was that online formative OSCEs were an excellent complement to on-site OSCEs. Teachers were also keen to participate in the future online OSCEs sessions.

Discussion:

Few studies have reported online OSCEs training in response to the COVID-19 pandemic [12,14,15]. The medical school of Université Paris Cité is the first to our knowledge to have organized large-scale and simultaneous eOSCE training, including 531 students and 270 teachers. Previous studies described virtual OSCEs in different health science disciplines as pharmacy and dentistry, and they were mainly designed as mock exams [12,14,16–18]. However, other studies conducted in Singapore[4], in the UK[19], or in Spain [20] reported virtual OSCEs as a qualifying exam for undergraduate medical students. Shaban et al. reported an experience on Microsoft Teams and developed and OSCE Time Management Dynamic Website, they set up each student in an individual virtual room on Teams for the entirety of the exam, and had examiners, simulated patients (SPs) and proctors rotating between them [21].Whereas Kakadia et al. reported eOSCES on 34 students and recommended using several Zoom links instead of breakout rooms and moving the teachers to reduce technical errors [16]. Therefore to ensure the ability to organize a large-scale OSCEs, we have developed a dedicated eOSCE application” with Zoom, which makes it possible to mass create numerous Zoom meetings from a CSV file containing teachers’ and students’ identities and contact information. With the eOSCE application development, our approach resolves this issue and is under further development with an ERASMUS+ funding to allow its release to other universities.”

As far as we know, this is the first time that such an application has been specifically developed to use Zoom for eOSCEs.

Our study confirms other reports’ findings that organizing such training is feasible but time consuming for teachers, administration, and IT teams [4,15,21]. Mock sessions must be scheduled to test the techniques [11]. Teachers’ training sessions are also mandatory to explain every Zoom function that they must master during eOSCEs, from waiting room management, video, and sound management to screen sharing and record functions. Our organization has shown that the SOS room could help teachers and students with any technical issues during the session. Therefore, we strongly advise considering the use of an SOS room while planning eOSCEs. This study also highlights that eOSCEs are helpful to assess several clinical skills such as history-taking, educational, laboratory results interpretation and communication skills but do not readily evaluate physical examination or procedural skills. Our report also confirms that both students and teachers expressed high satisfaction rates after this experience and appreciated the flexibility offered by eOSCEs. Mak et al. also reported a reduced level of stress among pharmacy students during online OSCEs, which can be explained by the fact that students are in the comfort of their homes and therefore avoid peer anxiety [18]. Furthermore, eOSCES are an appropriate response to social distancing measures and allow us to pursue medical students training; they also can be an excellent tool to prepare our students for telemedicine practices [17,18,22,23]. Telehealth use has increased within the last two decades, specifically during the COVID-19 pandemic[24]. While it was initially designed to facilitate access to the healthcare system in medical deserts, telehealth has reduced waiting times and travel times[25]. Medical student programs still lack training for telehealth practices[26], and eOSCEs might be a suitable tool to help them train for their future practice that might include telehealth.

### Limits

The absenteeism rate of 33% was significant since it was a mock exam and students’ participation was optional. This absenteeism may also have introduced a volunteer bias by selecting mostly technically-savvy students. Only half of the students participated in the video online debriefing, and less than half answered the online survey. The same bias might be considered for the teachers, although there were a higher proportion of them that participated in the debriefing and the online survey. Thus, the conceptual framework proposed here still needs to be evaluated in other contexts and university settings. In addition, eOSCEs on Zoom required almost as many staff members, including teachers and IT engineers, as on-site OSCEs. Finally, they can only be considered a training method complementary to on-site OSCEs rather than a sanctioning exam due to the risk of uncontrolled communication between the students during the evaluation, mainly when changing stations. Our pedagogical team is now studying several options to reduce the risk of communication between students and cheating during eOSCEs, such as the browser lockdown system, making them unable to open other internet browser windows during the eOSCEs, or the use of multiple versions of the stations by the simulated patients. All these limitation are currently being tackled within the ERASMUS+ project with aim to provide an easy to use platform available for other countries.

### Strengths

Both teachers and students had positive feedback and considered eOSCEs on Zoom as complementary to on-site OSCEs.

The eOSCE application development made it possible to simplify the process of mass creating Zoom meetings, and the application could be helpful for other educational purposes. This application is actually tested at the European level as part of an ERASMUS+ project.

Developing eOSCEs could promote OSCE training exchanges between medical schools in the same country and internationally. Furthermore, eOSCEs are also suitable for recording, thus presenting the possibility of post-evaluation and contradictory evaluations between several examiners. All the stations can be recorded on Zoom, which also allows for the possibility of a remote evaluation or a double evaluation. Our method allowed all those points and was straightforward to use by the students and the teachers on a vast scale. Indeed, as several other platforms, Zoom is now well known by all the students and teachers and allows for the possibility of sessions recording into a centralized system.”

## Conclusion

Organizing large-scale eOSCEs on Zoom is feasible with the appropriate tools and makes it possible to assess numerous skills, such as history taking, treatment strategies, patient information, or communication. Both teachers and students had positive feedback and were looking forward to future training sessions. Finally, eOCSEs on Zoom are still as time consuming as on site OSCEs and should be considered complementary to on-site OSCES and as a way to train medical students in telemedicine.
